# Utilising common bean and strawberry vegetative wastes in yellow mealworm (*Tenebrio molitor*) substrates: effects of pre-treatment on growth and composition

**DOI:** 10.1038/s41598-025-91732-3

**Published:** 2025-03-05

**Authors:** Wael Yakti, Simon Schulz, Nadja Förster, David Deruytter, Marcus Müller, Inga Mewis, Christian Ulrichs

**Affiliations:** 1 Urban Plant Ecophysiology Division, Faculty of Life Sciences, Thaer-Institute of Agricultural and Horticultural Sciences, Humboldt-Universität zu Berlin, 14195 Berlin, Germany; 2 Inagro, Insect Research Centre, 8800 Rumbeke-Beitem, Belgium

**Keywords:** Entomology, Environmental biotechnology, Zoology, Environmental sciences

## Abstract

Integrating plant production with insect farming, termed "entomoponics," involves using plant waste as a substrate for insect rearing, while returning insect frass to fertilise the plants. In this study, vegetative wastes from strawberry (*Fragaria* x *ananassa*), and common bean (*Phaseolus vulgaris*), were incorporated into a wheat bran-based substrate for rearing the yellow mealworms (MW; *Tenebrio molitor*). The wastes were either autoclaved or autoclaved then fermented with the fungus *Trichoderma reesei*, and mixed in a 50:50 ratio with wheat bran. Replacing 50% of the wheat bran with autoclaved beans waste did not significantly affect MW yield, but the yield was reduced when beans wastes were fermented or left untreated. Incorporating beans waste, whether treated or untreated, increased the Ca, K, and Fe content in the MW. Incorporating strawberry vegetative waste into the substrate compensated the yield regardless of the pre-treatment, but enhanced Mn, Zn, and Fe levels in the produced MW. Plant flavonoids were reduced when the wastes were pre-treated and did not accumulate in the MW biomass. These findings provide insights into using plant vegetative wastes as a partial supplement in MW rearing substrates, and the potential effects on the growth and nutritional composition of the resulting MW biomass.

## Introduction

The worldwide need for nutritionally balanced food is steadily increasing, given projections indicating that the global population may reach 9.5 billion by 2050^1,2^. In the year of 2017, global protein consumption reached around 202 million tons with meat and cereals being the primary sources^3^. In the case of meet, global demand is increasing and projected to reach 435 million tons by the year 2050^4^.

Given resource limitations and the growing effects of climate change, there is a rising need for circularity in food production systems to adopt maximal utilisation of available resources and re-use wastes^5^. The transition to circular economy necessitates this shift from conventional linear production towards circularity, allowing more sustainable production practices in today’s environmental context^5–7^.

The emergence of insect biorefinery as a waste management and protein production strategy has gained global attention^8,9^. With this approach, low value wastes can be fed to insects that can be later used as food and feed. Insect farming creates value from waste, generates economic benefits, and enhances sustainability in agriculture^10–12^, while reducing the environmental impact linked with waste disposal^13,14^. In addition to the use of insects biomass as food and feed, insects production generates a rest substrate “frass” that is rich in plant available nutrients and can be used as a fertiliser^15,16^, thus reducing synthetic fertilisers use and promoting environmentally friendly agricultural practices. A wide spectrum of organic wastes can be used as insects rearing substrates^17–20^ including plant leaves “green wastes” generated from plant production systems^11,12,21^. The coupling of plant production and insects production have been discussed in literature and termed as “entomoponics”^11,12^. The development and implementation of such a system necessitates more knowledge about the use of different plant vegetative wastes as feed for insects.

The yellow mealworm (MW) *Tenebrio molitor* (Coleoptera: Tenebrionidae) is a commonly farmed insects worldwide, and is considered safe for human consumption in the EU^22^, making it a higher value product compared to insects farmed for feed. MW offer nutritional advantages over other farmed insect species such as their high protein content and favourable amino acid composition^23,24^, and their high level of unsaturated fatty acids, vitamins, and minerals^25^. From the production point of view, the automation of MW big-scale production is arguably more efficient than many other insect species as they can be housed in the same “crates” system during the entire lifecycle, and their consumption of a dry substrate simplifies their sieving and processing^26^.

The insect is a common stored-products pest^27^, and can recycle wastes of animal and plant origins^28^. In this study, we tested the vegetative residues of strawberry (*Fragaria x ananassa* Duch.) and common bean (*Phaseolus vulgaris* L.) as partial replacement to the high-value wheat bran rearing substrate. The two waste streams were tested without pre-treatment, after autoclaving, or after solid-state fermentation with the fungus *Trichoderma reesei* “DSM 768”*.* The fungus *T. reesei* was chosen as it has been shown to enhance the nutritional value of waste streams when used to ferment insects substrates^29,30^*.* We hypothesised that the MW will utilise the plant residues and that the solid-state-fermentation with *T. reesei* will enhance their nutritional value.

## Results

**Fig. 1:**
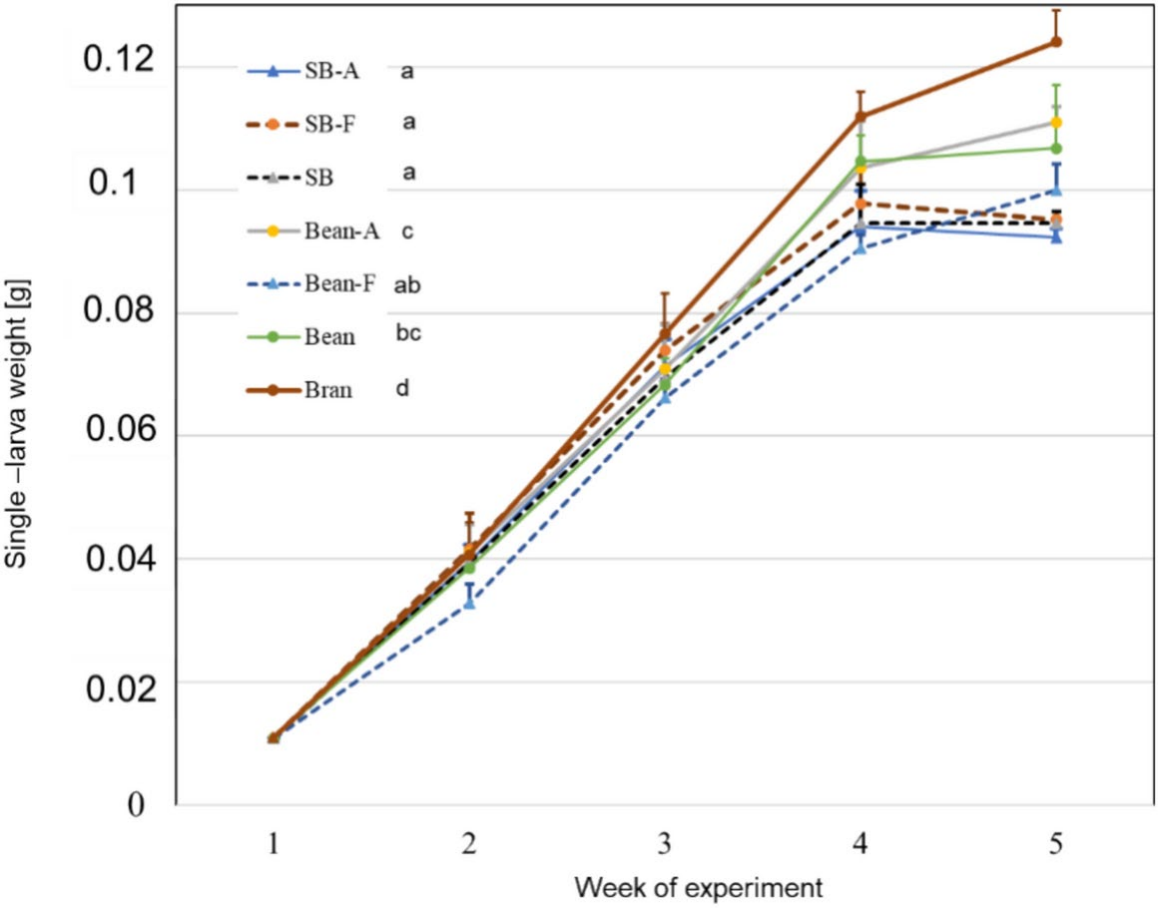
The growth of mealworm (MW) larvae. MW were grown on wheat bran (Bran). 50% of the wheat bran was substituted with autoclaved strawberry vegetative waste (SB-A), strawberry vegetative waste fermented with *T. reesei* (SB-F), untreated strawberry vegetative waste (SB), autoclaved beans vegetative waste (Bean-A), beans vegetative waste fermented with *T. reesei* (Bean-F), or untreated autoclaved beans vegetative waste (Bean). Shown are the mean single larvae weight with the standard deviations. One-way ANOVA (n = 5, *p* < 0.05) followed by Tukey’s test revealed significant differences in the single-larva weight on the day of harvest (5th week). Significant differences are represented by the different letters beside the legends.

The MW grew in all the boxes and MANOVA revealed a significant effect of the substrate on the combined production variables: single-larva weight at the day of harvest, fresh yield, dry yield, total dry frass produced, mortality, and pupation rate, but not on the feed conversion ratio (FCR) (Pillai’s Trace value = 2.86, F = 3.51, p < 0.001), and the separate one-way ANOVA revealed similar significant differences. The mean single-larva weight differed (ANOVA, n = 5, *p* < 0.05, F = 27.4), with values ranging from 92 to 124 mg at the time of harvest (Fig. 1).

The highest single-larva weight was observed in the wheat bran treatment. Replacing 50% of the wheat bran with autoclaved, untreated, or fermented beans waste led to significantly lower mean larva weight. Autoclaving the bean vegetative waste without fermenting lead to a higher single-larvae weight compared to the *T. reesei*-fermented bean waste. The lowest growth was observed in the treatments with strawberry waste, and the three treatments (autoclaved, fermented, or untreated) did not differ (Fig. 1).

**Fig. 2:**
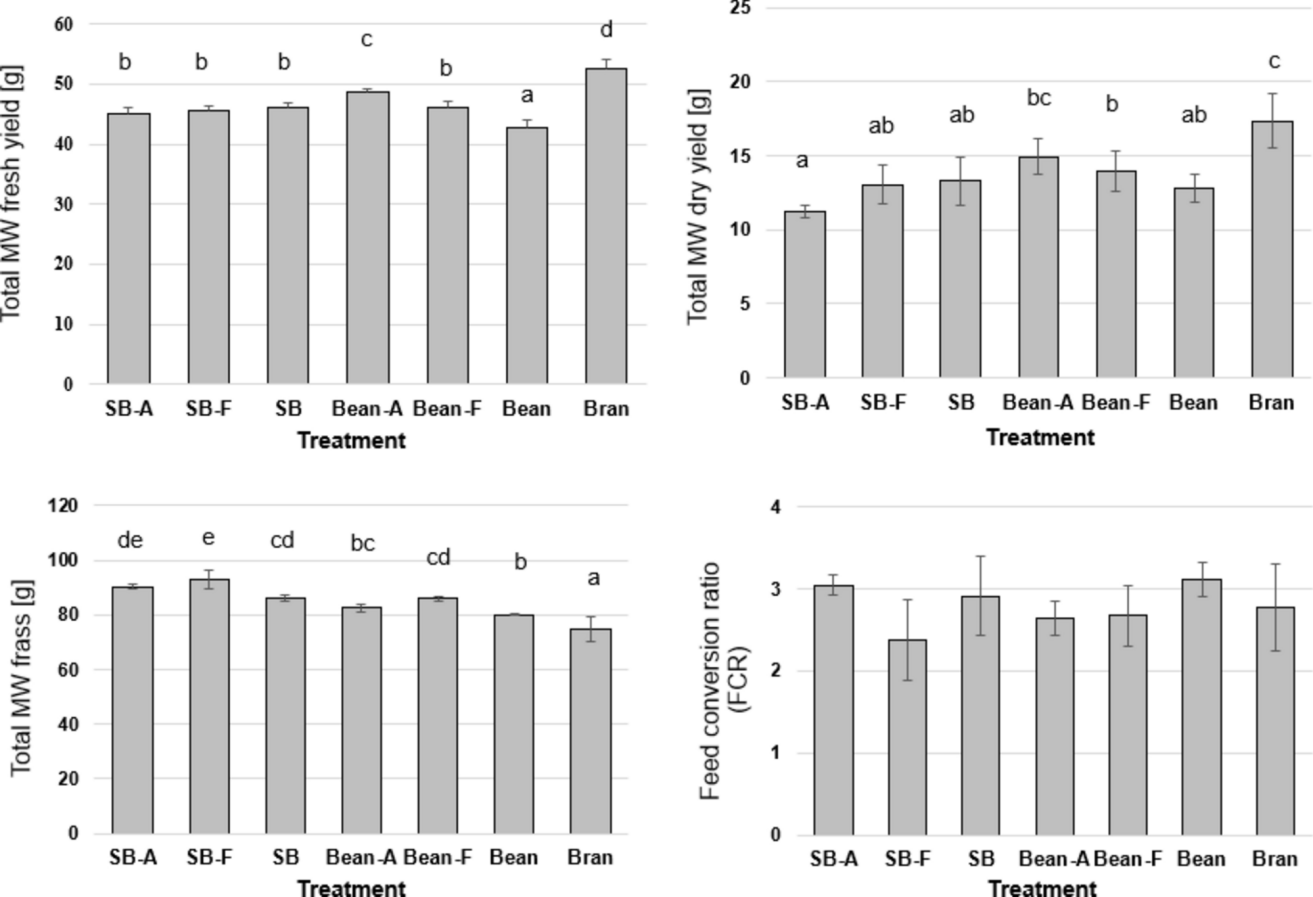
The total mealworms (MW) fresh yield, dry yield, total remaining frass, and the feed conversion ratio (FCR). MW were grown wheat bran (Bran). 50% of the wheat bran was substituted with autoclaved strawberry vegetative waste (SB-A), strawberry vegetative waste fermented with *T. reesei* (SB-F), untreated strawberry vegetative waste (SB), autoclaved beans vegetative waste (Bean-A), beans vegetative waste fermented with *T. reesei* (Bean-F), or untreated autoclaved beans vegetative waste (Bean). Shown are the mean values and standard deviations for the total dry yield, total weight of frass, and feed conversion ratio (FCR). Significant differences revealed by ANOVA followed by Tukey’s test (n = 5, *p* < 0.05) are represented by the different letters above the columns.

The fresh yield of MW varied among treatments (ANOVA, n = 5, *p* < 0.05, F = 44.8) and was the highest in the MW that received 100% wheat bran and the lowest when 50% of the wheat bran was replaced by the autoclaved strawberry leaves (Fig. 2). A comparable trend was observed in the total dry yield with significant differences among treatments (ANOVA, n = 5, *p* < 0.05, F = 10). Replacing 50% of the wheat bran with autoclaved beans waste did not lead to lower total dry yield, but the yield was reduced when the bean waste was fermented or untreated (Fig. 2). The inclusion of strawberry leaves led to significantly lower dry yield in comparison to the bran treatment. The treatments also differed in the consumption of substrate, leading to significant differences in the amount of remaining frass (ANOVA, n = 5, *p* < 0.05, F = 36.6). The amount of frass was the lowest in the MW that received wheat bran (Fig. 2), and the partial replacement of wheat bran significantly increased the amount of frass. However, no significant differences in the FCR were found among the treatments using both ANOVA and MANOVA tests.

**Fig. 3:**
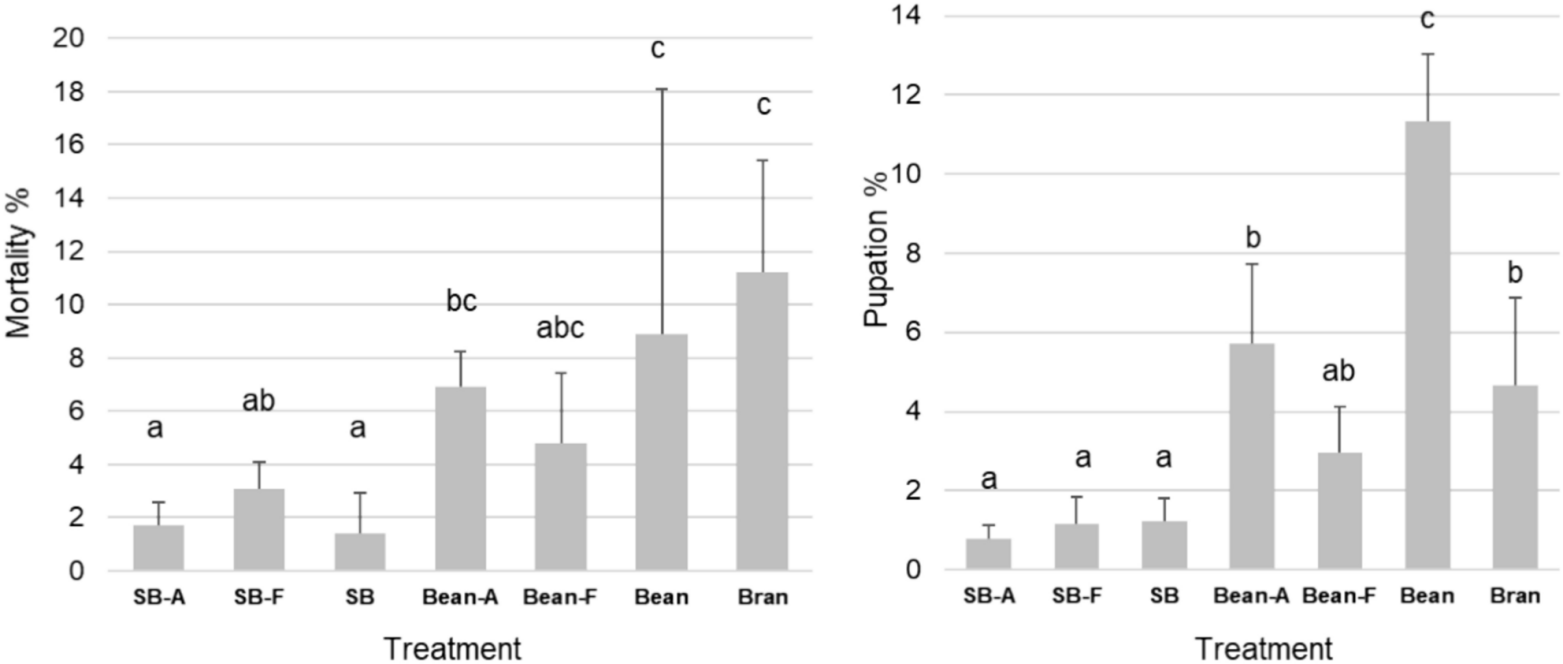
The mortality and pupation rate of mealworms. MW were grown on wheat bran (Bran). 50% of the wheat bran was substituted with autoclaved strawberry vegetative waste (SB-A), strawberry vegetative waste fermented with *T. reesei* (SB-F), untreated strawberry vegetative waste (SB), autoclaved beans vegetative waste (Bean-A), beans vegetative waste fermented with *T. reesei* (Bean-F), or untreated autoclaved beans vegetative waste (Bean). Shown are the mean values and standard deviations. Significant differences in pupation rate were revealed using ANOVA followed by Tukey’s test (n = 5, *p* < 0.05), and in Mortality using Kruskal–Wallis and Dunn’s tests (n = 5, *p* < 0.05). The significant differences are represented by the different letters above the columns.

The highest mortality was recorded for MW that received the wheat bran (control) and when 50% of wheat bran is replaced by the beans waste variants, without observed significant differences among these treatments. However, the inclusion of untreated or autoclaved strawberry significantly reduced the mortality in comparison to the control, the untreated bean wastes, and the autoclaved bean wastes treatments (Kruskal–Wallis-Test, n = 5, *p* > 0.05, H = 22.1) (Fig. 3). The pupation rate of MW also significantly differed among the treatments (ANOVA, n = 5, *p* > 0.05, F = 34.4) and was the highest when 50% of the wheat bran in the MW substrate were replaced by untreated beans wastes. The inclusion of strawberry leaves into the substrate lead to the lowest pupation rate (Fig. 3).

The partial elemental analysis of the produced mealworms revealed significant differences in Ca, P, Mg, K, S, Cu, Zn, Mn, and Fe values, without significant differences in the crude protein content in the MW (Table 1). The inclusion of beans vegetative waste, regardless of the pre-treatment, enriched the MW biomass with Ca, Zn and Fe, while reducing the Cu content. Additionally, replacing 50% of the wheat bran with the strawberry vegetative waste increased the P, Fe, and Zn. K content was enriched in the MW biomass with the inclusion of strawberry leaves regardless of the pre-treatment.

**Table 1:** The partial composition of the produced MW biomass.

Treatment		Protein [%]	Ca [%]	P [%]	Mg [%]	K [%]	S [%]	Cu [mg/kg]	Zn [mg/kg]	Mn [mg/kg]	Fe [mg/kg]
SB-A	Mean	36.86	0.19^ab^	1.046^b^	0.25^a^	1.67^a^	0.31^a^	16.63^ac^	120.33^a^	26.37^a^	114.33^a^
	sd	0.3	0.01	0.006	0.006	0.010	0.001	0.15	0.58	1.72	6.66
SB-F	Mean	37.08	0.14^a^	1.06^b^	0.27^a^	1.72^a^	0.31^ab^	17.2^ac^	122.66^a^	13.9^ab^	61.63^de^
	sd	2.25	0.006	0.062	0.010	0.099	0.023	1	7.23	0.7	3.85
SB	Mean	36.89	0.16^ab^	0.96^ab^	0.24^a^	1.61^a^	0.31^ab^	16.56^ac^	118.33^ac^	20.56^a^	104.13^ab^
	sd	1.05	0.006	0.021	0.012	0.040	0.010	0.65	4.04	0.7	6.02
Bean-A	Mean	37.58	0.38^c^	0.83^ab^	0.2^a^	1.33^b^	0.34^ab^	14.97^bc^	162^b^	15.63^a^	89.033^bc^
	sd	0.64	0.036	0.025	0.01	0.06	0.006	0.75	5	0.75	9.5
Bean-F	Mean	38.19	0.26^b^	0.7^a^	0.17^b^	1.18^bc^	0.33^ab^	13.8^b^	144^ab^	11.47^b^	76.4 cd
	sd	1.04	0.026	0.015	0.006	0.03	0.006	0.500	8.185	0.115	4.62
Bean	Mean	40.07	0.25^b^	0.69^a^	0.17^b^	1.14^c^	0.34^ab^	13.36^b^	152.66^ab^	12.83^b^	66.4^de^
	sd	0.52	0.015	0.021	0.006	0.064	0.012	0.611	2.517	0.764	3.26
Bran	Mean	36.17	0.02^a^	0.79^a^	0.23^a^	1.06^c^	0.29^a^	17.53^a^	112.67^c^	12.9^b^	51.06 f.
	sd	0.31	0.01	0.01	0.01	0.015	0.002	0.153	0.577	0.6	1.553
*p*		0.065	0.004	0.004	0.004	< 0.001	0.016	0.01	< 0.001	0.004	< 0.001
H value (Kruskal–Wallis test)		11.86	19.40	19.00	18.90		15.60	17.30		18.89	
F value (ANOVA)						81.19			46.80		51.11

MW were grown on wheat bran (Bran). 50% of the wheat bran was substituted with autoclaved strawberry vegetative waste (SB-A), strawberry vegetative waste fermented with *T. reesei* (SB-F), untreated strawberry vegetative waste (SB), autoclaved beans vegetative waste (Bean-A), beans vegetative waste fermented with *T. reesei* (Bean-F), or untreated autoclaved beans vegetative waste (Bean). Significant differences for each element are expressed with different letters beside the mean values (n = 3, *p* < 0.05).

Three flavonoids were identified in the strawberry waste used in the MW feeding experiments (SB, SB autoclaved, and SB fermented; Table 2), none of which were detected in the MW fed on these substrates. However, a kaempferol derivate not found in the substrates was detected in the MW fed on the fermented strawberry waste (SB-F). The results of the beans waste were similar, showing a higher diversity of flavonoids (six) in the untreated beans waste, and forming new quercetin derivates in the MW produced on the untreated beans waste and the autoclaved bean waste (Bean and Bean-A, respectively). The autoclaved and fermented strawberry waste differed significantly in their total flavonoid content (t-test, n = 3, *p* < 0.05, F = 281.3), and had lower values compared to the untreated strawberry waste. In the case of beans waste, the drop in the total flavonoid content was observed between autoclaving and fermenting the waste (t-test, n = 3, *p* < 0.05, F = 14) (Table 2). In the produced MW biomass, however, the flavonoid contents were lower compared to the corresponding feeding substrate. No flavonoids could be detected in the MW that received fermented strawberry waste (SB-A), the untreated strawberry waste (SB), or the fermented bean waste (Bean-F) (Table 2). In the wheat bran material used as control for the feeding experiments no flavonoids could be detected. Consequently, in the larvae fed on this substrate no flavonoids could be detected either.

**Table 2:** Flavonoid contents in [µmol/g DW] of the feeding substrates and the MW produced.

Flavonoid content	Feeding substrates			MW produced		
	SB	SB autoclaved	SB fermented	SB-A	SB-F	SB
Quercetin glucuronide	9.32	1.38	0.25	0	0	0
Kaempferol-glucoronides	0.65	1.03	0.16	0	0	0
Kaempferol coumaryl hexoside	0.53	1.02	0.58	0	0	0
Kaempferol derivate	0	0	0	0	0.04	0
Total	10.50	3.43^a^	0.99^b^	0	0.04	0

Flavonoid content	Feeding substrates			MW produced		
	Bean	Bean autoclaved	Bean fermenteddF	Bean-A	Bean-F	Bean
Quercetin derivate	1.18	1.22	0.82	0.36	0	0.26
Quercetin derivate	0	0	0	0.10	0	0.07
Kaempferol-dihexoside-hexoside	0.32	0.31	0.21	0.08	0	0.05
Quercetin-sophorotrioside-glucoside	0.28	0.28	0.23	0	0	0
Quercetin-sinapoyl-sophorotrioside	1.03	0.79	0.47	0	0	0
Luteolin derivate	13.43	13.30	5.91	0	0	0
Kaempferol-dihexoside-hexoside derivate	2.36	2.21	1.12	0	0	0
Luteolin derivate	0.73	0.63	0.26	0.04	0	0.05
Total	19.33	18.73^a^	9.01^b^	0.59	0	0.44

Feeding substrates: untreated strawberry and common bean vegetative waste (SB/Bean), autoclaved vegetative waste (SB autoclaved/Bean autoclaved), or vegetative waste fermented with *T. reesei* (SB fermented/Bean fermented). MW produced: MW grown on untreated vegetative waste (SB/Bean), autoclaved vegetative waste (SB-A/Bean-A), or vegetative waste fermented with *T. reesei* (SB-F/Bean-F). Significant differences in the total flavonoid content are indicated by different letters.

## Discussion

Beans are widely recognised as one of the finest plant-based sources of high-quality protein, ranging from 16 to 33% in content depending on the variety^31^, and common bean can account for more than 80% of worlds beans production^32^. The post-harvest residues of common beans (*Phaseolus vulgaris* L.), comprising leaves and stems (straw), are abundant and freely accessible, and are typically discarded on farms after bean pods are harvested. These residues could offer an economic opportunity when utilised as livestock feed. Compared to barley and wheat straw, legume residues exhibit high levels of protein and energy^33^. The incorporation of bean leaves in animal diets has been studied on wistar male rats receiving high-fat/high-sugar diets and have been shown to reduce weight gain and fat accumulation, most likely due to the presence of bioactive compounds^34^. However, due to the high fibre content and potential antinutrients in the metabolite groups of tannins and phenolic compounds, only limited amounts of legume leaves could be integrated into animal diets and pre-processing is recommended for optimal integration^35–37^. Another abundant plant residue is the leaves and stems of strawberry with a limited number of studies assessing their use as animal feed. Strawberry vegetative wastes are suitable as a partial replacement for clover hay in rabbit diets, leading to enhanced growth performance and economic efficiency^38^. Strawberry leaves also contain bioactive secondary metabolites such as tannins, flavonoids, and ascorbic acid^39^ enhancing their bioactivity.

The vegetative waste of strawberry have been only reported in a production system as a moist supplement for MW^40^, without emphasis of its nutritional value. To the best of our knowledge, the presented study is the first to test the dried beans and strawberry vegetative wastes as supplement in MW feeding substrates.

The elemental composition of the vegetative plant biomass can depend on many factors including the plants’ access to nutrients in a cultivation system (e.g. hydroponic vs. soil-grown)^41^, seasonal changes^42^ or stress^43^. The elemental composition of the strawberry vegetative waste used in this study was in the range observed in strawberry leaves by Dominguez, et al.^42^ for K, Ca and Zn, while they were either above or below for other elements such as N, Mg, and Fe. The bean vegetative waste used in our study had a relatively high N, K, S and Ca content, and the concentration of all elements were higher than those described by Oyelude, et al.^44^. Tuma, et al.^45^ reported differences in the K, Mg and Ca values among different beans plant parts and based on nutritional conditions. In the present study, the total plant shoots (including stems and leaves, excluding fruits/pods) were combined and homogenised for both bean and strawberry waste streams. This approach acknowledges that the composition may differ from that of a certain plant material reported in literature, in addition to various factors affecting the elemental makeup of the wastes. Consequently, it’s crucial to evaluate the composition of plant waste (as well as waste streams in general) on a case-by-case basis.

In this study, the two waste streams were pre-processed (autoclaved and fermented) and used to substitute 50% of the wheat bran in MW feeding substrate. The highest single-larva weight was observed in the 100% wheat bran treatment, and the growth was compensated with the inclusion of plant waste (Fig. 1). Differences in MW growth parameters were also observed as the MW fed with the autoclaved beans waste performed better than the waste fermented with *T. reesei*, and better than the strawberry waste variants (Figs. 1 and 2).

Despite the big body of literature on the use of food wastes and high quality substrates in MW feeding substrate^46^, only few studies have investigated the inclusion of dry plant vegetative wastes into MW substrate probably due to their high fibre content, relatively low macronutrients content, and the potential antinutrients. A study by Harsányi, et al.^47^ investigated the inclusion garden wastes into MW rearing, and the wastes contained various components such as grasses, tree leaves, and stone fruits which could provide sufficient macronutrients and have led to a better MW performance in comparison to normal vegetable wastes. Incorporating *Moringa oleifera* leaves into the diet of MW has been shown to elevate their nutritional quality without reducing growth, in addition to boosting crude protein and vitamin levels. This was observed even at 50% inclusion rate^48^. In a study by Bai-wen, et al.^49^, walnut leaves were incorporated into a wheat bran- based rearing substrate and lead to a reduced MW growth unless the leaves were fermented. Leaves of trees such as *Punica granatum, Castanea sativa*, and *Robinia pseudoacacia* have been shown to fortify MW biomass when incorporated at 10% ratio in MW feeding substrate^50^. The bean vegetative wastes in this study contained higher N levels than both the wheat bran and the strawberry leaves (Table 3), and the reduced growth could be explained by the expected lower energy content in comparison to the control bran. However, the final yield was not compensated when 50% of the wheat bran was replaced by autoclaved bean vegetative waste (Fig. 2).

**Table 3:** The partial elemental composition of the wheat bran and the two waste streams used in the MW feeding experiment.

	N [%]	Ca [%]	P [%]	Mg [%]	K [%]	S [%]	Cu [mg/kg]	Zn [mg/kg]	Mn [mg/kg]	Fe [mg/kg]
Wheat bran	2.55	0.14	1.13	0.44	1.52	0.19	11.3	88	115	200
Beans waste	2.66	2.3	0.27	0.28	3.13	0.57	5.2	77.4	36.8	151
Strawberry waste	1.16	1.44	0.2	0.29	1.93	0.09	10.3	32	133	1070

Subjecting plant materials to autoclaving or heat treatment could significantly alter their nutritional composition, notably impacting fibers properties and composition, along with various other nutritional and anti-nutritional factors. For example autoclaving beans decrease the activity of trypsin and α-amylase inhibitors, which enhances nutrient bioavailability of both protein and carbohydrates^51^. Comparable effects could be anticipated for bean vegetative waste when autoclaved. The outcome of thermal pre-treatment of substrates relies on temperature, duration, pressure and probably the target insect species. For instance, in a study by Isibika, et al.^30^, a 60-min heat treatment of banana peels at 120 °C and 2 bar lead to decreased growth and conversion rate of black soldier fly larvae (BSFL) attributing this effect to the release of toxic tannins. In a study on the sewage sludge, 16 h thermal treatments at 90 °C boosted BSFL growth and lead to the highest protein and fat content in the BSFL biomass^52^. Besides changing the bioavailability of nutrients and removing antinutrients, thermal treatments can also decrease or eradicate microbial activity, which could have either positive or negative outcomes on insect growth^53^.

We hypothesised that the solid-state-fermentation (SSF) with *T. reesei* would boost the nutritional value of the plant wastes tested in this study. Developing solid-state fermentation (SSF) techniques aimed at improving nutrient accessibility for insects holds the potential to improve their performance on high-fibre low-value wastes, in addition to modulating the secondary metabolites profile of the substrates, and consequently the produced insect biomass^54^. The enzymatic arsenal *T. reesei*, especially the enzymes involved in the cellulolytic activity, can play a crucial role in converting lignocellulosic biomass into valuable feed ingredients^29,55^. *T. reesei* has been used to ferment banana peels^30^ and trimmings of brassica plants^56^ leading to enhanced nutrient availability, thereby improving the bioconversion process of black soldier fly larvae (BSFL). In the current study, however, the fermentation with *T. reesei* did not enhance the growth of MW on the two tested plant materials (Figs. 1 and 2) and lead to negative outcomes in comparison to the autoclaved bean vegetative waste. This could be, partially, due to the production of fungal antinutritional secondary metabolites such as trichocellin A-I and B-II which have potential insecticidal activity^57^. This, however, did not lead to a higher mortality MW in the fermented treatments, but could have hindered the growth of MW in the presented experiment. Flavonoid contents were drastically reduced for strawberry (autoclaved and fermented) and bean (fermented) substrates in the present study (Table 2), demonstrating the big impact of pre-treating substrates on the flavonoid profile of substrate.

The results of both Multivariate (MANOVA) and Univariate (ANOVA) aligned indicating that each production variable responds independently to the substrate factor/treatment with low correlation between the production parameters as a whole. In both tests, the differences observed in MW yield among treatments did not translate to differences in the feed conversion ratio (FCR) (Fig. 2). Studies on MW indicate that altering substrates and environmental conditions can impact their growth without necessarily leading to changes in the FCR. MW given substrates supplied with fresh plant materials such as carrot, orange, or red cabbage showed enhanced growth rates with survival, while feed conversion stayed unaffected^58^. In this study, the FCR values stayed unchanged due to the lower consumption of substrate in the treatments with plant waste treatments, which also explains the higher frass amounts (Fig. 2) included in calculating the FCR. The frass amount was high in these treatments likely due to the high fibre content in the plant vegetative waste.

The mortality of MW in the present experiment differed among the treatments and was the highest in the MW receiving 100% wheat bran and the MW receiving 50% untreated beans waste (Fig. 3). A study by Zim et al.^59^ have found that replacing wheat bran with citrus and tomato crop residues leads to a very high mortality, which can be explained by presence of insecticidal plant secondary metabolites. Despite the differences observed, the mortality values in the present study can be considered low (< 12%) and comparable to other studies that tested non-toxic substrates^21,60,61^. Nevertheless, the level of protein and carbohydrates in the MW feeding substrate could have led to differences in mortality, as emphasised in previous studies^62,63^. According to Kröncke and Benning^64^, MW exhibit a preference for grain-based diets such as wheat bran, flour, maize hulls, and oat bran, and this preference indirectly impact their development and pupation rates. However, in the present study the inclusion of untreated bean wastes led to a higher pupation rate compared to the 100% wheat bran substrate. The influence of diets on the development of MW is acknowledged in the literature^47,65^, and understanding the specific mechanisms that lead to the differential pupation rate is a topic for future research.

The elemental composition of MW has been shown to be influenced by the composition of the substrates they consume^21,66^. The values of Mg, K, Cu, Zn, Mn, and Fe are comparable to those obtained in other studies^67,68^. In the current study, the concentration of Ca and P in the MW did not seem to correlate with the concentration in the substrate component added (Table 2). MW have been shown to contain less Ca compared to other insects such as the black soldier fly^21^ and the enrichment of Ca in the MW biomass through gut loading has been reported by Boykin, et al.^69^ as an approach to increase Ca content. The incorporation of beans waste fortified the MW with Ca which can be considered an added value. Such fortification was also observed when incorporating hemp waste into MW feeding substrate^21^. An accumulation of flavonoids in the MWs was not observed. Here, traces could be detected in the MW biomass regardless of the feeding substrate. In some cases, flavonoids were detected in the MW that were not found in the substrates (kaempferol derivate in SB-F, quercetin derivate in Bean and Bean-F; Table 2). This indicates a possible formation of decomposition products, which can easily occur in complex flavonoid depending on their chemical structure, for example via glycosylation or hydroxylation^70^. Additionally, in this experiment the waste materials were pre-processed by drying at 60 °C to mimic production scenarios, which is not optimal to conserve a high flavonoid content in the plant material.

Thermal processing (autoclaving or cooking) drastically changes the accessibility of nutrients in foods^71^, and this was the case in this study as the concentration of Mn, Fe, K, Mg, and Ca in the MW increased when the beans vegetative waste was autoclaved. The integration of plant wastes in the substrate has led to higher Fe, P and Zn in the MW despite the lower concentration in the initial substrate which can improve the value of the MW biomass and boost their nutritional value^72^.

As a conclusion, this study aimed to test the integration of vegetative strawberry and common beans waste into MW rearing substrate, in addition to testing the influence of pre-treating these wastes (solid-state fermentation with the fungus *T. reesei* and/or autoclaving). The lowest MW growth was observed when wheat bran was partially replaced by strawberry vegetative waste regardless of the pre-treatment. Common beans vegetative waste lead to better MW growth, as replacing 50% of wheat bran with autoclaved common beans waste did not decrease final dry MW yield. The utilisation of plant vegetative wastes as MW feeding substrates is an approach to minimise the costs associated with the substrates and can produce fortified larvae with improved nutritional composition. This can be cost efficient especially in regions where drying can be sustainable and free (e.g. sun drying). However, more research is needed to identify the substrate characteristics that change with pre-treatments leading to an enhanced larval yield. Additionally, the accumulation of plants associated antinutrients in the larvae (e.g. phytic acid) has to be investigated to ensure product safety and applicability.

## Methods

### Yellow mealworms

*Tenebrio monitor* (Coleoptera: Tenebrionidae) eggs were provided by the Insect Research Centre of Inagro (Rumbeke-Beitem, Roeselare, Belgium). 60 g of mealworm eggs were placed on 1.2 kg of wheat bran with a particle size lower than 2 mm (LM “Lindenberger mill” GmbH, Brandenburg, Germany). The mealworm (MW) were incubated for 5 weeks at 28 °C and 40–60% RH, with a constant provision of 20% agar as a source of moist feed. The MW were manually sieved and a pool of larvae with an average weight of 10 mg was used in the experiments.

### Plant materials

Common beans seeds (*Phaseolus vulgaris* L. var. Modesto) were obtained from Kiepenkerl (Norken, Rhineland-Palatinate, Germany) and cultivated in open land at the Humboldt University in Berlin, Germany (52.467715° N, 13.298407° W). All produced bean pods were harvested from the plants and only the remaining vegetative parts (leaves and stems) were collected and used in the experiments. The strawberry leaves and stem wastes were obtained from Martin Bauer GmbH (Vestenbergsgreuth, Bavaria, Germany) and comprised of the vegetative parts gathered from various growth periods and diverse strawberry (*Fragaria × ananassa*) cultivars. Both waste streams were dried at 60 °C and processed into powder using Bergman ECOLINE food processor (Luchs AG, Bochum, North Rhine-Westphalia, Germany). The partial elemental composition of the two waste streams in addition to the used wheat bran are shown in Table 3.

### Solid-state fermentation

Distilled water was gradually added to the dried strawberry waste and the common beans vegetative waste, and the mixtures were stirred with a kitchen spatula until a wet porous substrate without any observable free water was formed having 26.8% dry matter content. After preparation, 150 g of each substrate was transferred to a 500 ml beaker and covered from the top with aluminium foil. The beakers were autoclaved at 121 °C for 20 min and were, thereafter, inoculated with *T. reesei* “DSM 768” by placing five plugs (7.5 mm in diameter) of the mycelium grown on PDA medium (Carl Roth GmbH + Co. KG, Karlsruhe, Germany) onto the top of the substrate. Additionally, autoclaved beakers were kept uninoculated. From each plant material, 5 inoculated and 5 non- inoculated beakers (n = 5) were incubated at 26 °C for 3 weeks allowing the fungus to colonise and cover the inoculated substrates. After 3 weeks, samples were taken from 3 beakers after homogenisation for later analysis of flavonoids and dried at 60 °C. For the MW feeding experiments, the contents of all beakers of one treatment were pooled, homogenised, dried at 60 °C, and used for MW substrate preparation.

### Mealworm feeding experiment

A MW feeding experiment was carried out to test how including pre-treated and non-treated strawberry waste and common beans waste in mealworm feeding substrates would affect MW performance and composition. The experiment consisted of seven treatments with five replicates each (n = 5). Wheat bran (LM “Lindenberger mill” GmbH, Brandenburg, Germany) was used as a control substrate “Bran”, 50% of which was replaced by: 1. fermented strawberry vegetative waste “SB-F”, 2. autoclaved strawberry vegetative waste “SB-A”, 3. non-treated strawberry vegetative waste “SB”, 4. fermented beans vegetative waste “Bean-F”, 5. autoclaved beans vegetative waste “Bean-A”, or 6. non-treated beans vegetative waste “Bean”. The different dry substrates were prepared and homogenised in batches, then 120 g were put in 13 × 17 cm plastic containers in five replicates. 500 mealworms (with 10 mg mean weight, counted manually) were added on the top of the substrate and were let to grow for 5 weeks at 28 °C and 40–60% RH. Three plugs of 20 g/L agar (1 cm Ø and 0.5 cm high) were positioned on top of substrate *ad libitum*, ensuring that the larvae of all boxes consistently had access to the agar^73^. Unconsumed agar plugs, when remained, were regularly removed upon the provision of new plugs.

The growth of the MW was measured weekly by manually collecting and weighting at least 10% of the total number of larvae per box, and the MW were put back in the boxes after collecting the data. On the 5th week, all larvae were sieved, quantified and weighed, and the number of pupae was determined in addition to the amount of frass. After the harvest, the larvae were freeze-dried to obtain the total dry yield and to be later analysed. In addition to biomass data, the mortality rate and the feed conversion ratio (FCR) were calculated using the following equations:

$$Mortality\;rate\left( \% \right) = \frac{The\;initial\;number\;of\;MW - the\;number\;of\;MW\;at\;the\;time\;of\;harvest}{{The\;initial\;number\;of\;MW}} \times 100$$

$$FCR\left( {On\;dry\;basis} \right) = \frac{Intital\;weight\;of\;substrate \left( g \right) - Frass\;weight\left( g \right)}{{The\;weight\;gain\;of\;the\;larvae\left( g \right)}}$$

### Chemical analyses

The initial substrate components (wheat bran, strawberry waste, and common beans waste) and the produced MW (n = 3) were analysed for Ca, P, Mg, K, S, Cu, Zn, Mn, Fe, and N (for crude protein determination). The samples were grounded using coffee grinder (CLOER 7579, Arnsberg, Germany) and elemental analysis (excluding nitrogen) was conducted by microwave digestion using a MARS Xpress instrument (CEM, Matthews North Carolina), following LUFA methods Vol. III, 10.8.1.2. as described by Yakti, et al.^74^. The Multi-element analysis was performed using ICP-OES (DIN EN ISO 11885) and ICP emission spectrometer (iCAP 6300 Duo MFC, Thermo; Waltham, MA, USA). For Crude protein determination, total N was analysed using an elemental analyser (Vario MAX, Elementar Analysensysteme GmbH, Hanau, Germany) according to LUFA Bd. III, 4.1.2. The crude protein content of the MW was determined using a protein-to-nitrogen conversion factor of 4.76^75^. The flavonoids were analysed according to a method described by Förster, et al.^76^. Briefly, the waste or insect materials were subjected to extraction with 70% methanol (v/v; pH = 4, acetic acid). Flavonoids in the different extracts were quantified via HPLC, and identified based on their UV-spectra and mass spectrometry (ESI–MS, negative mode).

### Statistical analyses

All statistical analyses were carried out using SPSS version 28.0.0.0 software (IBM Corp, New York, NY, USA). To test the overall effect of the feeding substrate on multiple production variables simultaneously, a Multivariate Analysis of Variance (MANOVA) was performed using the single-larva weight at the day of harvest, fresh yield, dry yield, total frass produced, FCR mortality, and pupation rate as dependent variables. Thereafter, all measured parameters were compared using one-way Analysis of Variance (ANOVA) (n = 5–3, *p* > 0.05) followed by Tukey’s test after testing the normality and homogeny of variance. Kruskal–Wallis test and Dunn’s test for multiple comparisons were used for parameters that did not meet the assumption of parametric tests.

## Data Availability

Data sets generated and analysed during the current study are available from the corresponding author on reasonable request.
